# Neuromuscular adaptations to 4 weeks of intensive drop jump training in well-trained athletes

**DOI:** 10.1002/phy2.99

**Published:** 2013-10-16

**Authors:** Tine Alkjaer, Jacob Meyland, Peter C Raffalt, Jesper Lundbye-Jensen, Erik B Simonsen

**Affiliations:** 1 Department of Neuroscience and Pharmacology, University of CopenhagenCopenhagen, Denmark; 2 Department of Nutrition, Exercise and Sports, University of CopenhagenCopenhagen, Denmark

**Keywords:** Drop jump, motor learning, neuromuscular adaptations, performance, training

## Abstract

This study examined the effects of 4 weeks of intensive drop jump training in well-trained athletes on jumping performance and underlying changes in biomechanics and neuromuscular adaptations. Nine well-trained athletes at high national competition level within sprinting and jumping disciplines participated in the study. The training was supervised and augmented feedback on performance was used to ensure maximal training intensity. The drop jumps were performed with minimal contact time and maximal jumping height. Assessment of performance during training showed effects of motor learning. Before and after the training intervention maximal isometric muscle strength, the biomechanics, muscle activity pattern of the lower extremities and the soleus H-reflex and V-wave during drop jumping were measured. Maximal jump height and performance index (PI) defined as jumping height divided by contact time improved significantly by 11.9% (*P* = 0.024) and 16.2% (*P* = 0.009), respectively. Combined ankle and knee joint peak power was significantly increased by 7% after training (*P* = 0.047). The preactivity in the soleus muscle decreased 16% (*P* = 0.015). The soleus H-reflex was unchanged after training, while the soleus V-wave increased significantly at 45 msec after touchdown. This may indicate an increased drive to the α-motor neuron pool following training. Muscle strength parameters were unaffected by the training. The results demonstrate that 4 weeks of intensive drop jump training can improve jumping performance also in well-trained athletes without concomitant changes in muscle strength. It is suggested that the behavioral improvement is primarily due to neural factors regulating the activation pattern controlling the drop jump movement.

## Introduction

Several types of sport performance are highly dependent on fast stretch-shortening cycles in the muscles of the lower extremities. For example in track and field, volleyball, basketball, soccer, and badminton, the ability to generate explosive power is crucial to performance. To improve this quality athletes have used so-called plyometric training such as drop jumps, which implies jumping down from a plateau and on contact immediately reverse the movement to a maximal vertical takeoff (Schmidtbleicher et al. 1987; Young 2006; Markovic 2007). It has previously been demonstrated that drop jump performance is related to sprint running, triple jump, and high jump performance (Steben and Steben 1981; Holm et al. 2008; Kale et al. 2009). Without specific instructions other than aiming for maximal vertical jumping height, drop jumps are performed in at least two different ways: (1) fast, that is, with a very short contact time and (2) slower, that is, with somewhat longer contact time on the ground (Bobbert et al. 1986, 1987; Dyhre-Poulsen et al. 1991; Walsh et al. 2004). However, in many sport events time on the ground is highly limited in relation to jumping. In long jump the contact time on the takeoff board is ∼120 msec (Seyfarth et al. 1999) during which the muscles of the leg are required to go through a stretch-shortening contraction consisting of first an eccentric then a concentric contraction. Thus, it seems relevant to minimize the contact time in drop jump training. It has been shown that drop jump training may improve jump height (JH) within only 4 weeks of training in relatively untrained individuals (Schmidtbleicher et al. 1987; Bobbert 1990; de Villarreal et al. 2009; Taube et al. 2011). However, it is still unknown whether or not 4 weeks of intensive drop jump training performed with emphasis on very short contact time and maximal jumping height can improve jumping performance in well-trained athletes with a high level of jumping experience. Furthermore the mechanisms underlying improvements in performance are largely unknown. Thus, the purpose of the present study was to examine the effect of 4 weeks intensive drop jump training from a moderate drop height and performed with very short contact time on maximal jumping height and performance index (PI) defined as jumping height divided by contact time in a group of well-trained athletes. We hypothesized that it would be possible to improve jumping performance in well-trained athletes with a high level of jumping experience after 4 weeks of training and that this improvement would be caused by neural and not morphological changes due to the short training period. To test this claim we recruited a group of highly well-trained athletes who underwent 4 weeks of intensive drop jump training. The study subjects were highly skilled jumpers but none had drop jumping included in their daily training. In addition, comprehensive measurements were obtained before and after the training intervention in order to identify the biomechanical and neuromuscular factors underlying the expected improvements in jumping performance.

## Methods

### Subjects

Nine well-trained athletes (eight males and one female, mean [SD]: age: 24.4 [4.01] years, height: 1.80 [0.08] m, weight: 70.97 [8.63] kg) participated in the study. All the subjects had jumping experience (high national competition level within sprinting/jumping disciplines with >5 weekly training sessions), but none of them had drop jumps as a part of their daily training. Prior to participation, all subjects gave their written informed consent to participate in the experiments, which were approved by the local ethics committee (J. No. H-4-2010-107). The study was performed in accordance with the Helsinki II declaration.

### Training protocol

The study comprised three parts. A pre- and posttest separated by 4 weeks of drop jump training. The training period consisted of three weekly sessions. During the first and second week of training the subjects were exposed to 3 × 8 maximal drop jumps in each session, and during the third and fourth week they were exposed to 4 × 8 maximal drop jumps in each session. Except from one subject who did all his training on his own, the training sessions were supervised and feedback on each individual drop jump regarding performance was provided to the subjects. In total, 3024 drop jumps were performed during the training period of which 1848 drop jumps were supervised and 1447 jumps were recorded and used for evaluation of the jumping performance over the 4 weeks of training. It was not possible to monitor all training sessions since the athletes in some cases were prevented from performing their training at the laboratory. The reasons for this were primarily changes in the subjects’ daily schedules, transportation difficulties, and work situation. When the subjects performed their training on their own they were equipped with a small table adjusted to the individual drop height in order to ensure the correct drop jump training dose.

During the supervised training sessions the drop jumps were performed on a force platform. The athletes were provided feedback regarding their jumping performance, which was done trial by trial by immediate calculation of the JH and PI from the vertical ground reaction force. The feedback was given verbally by the principal investigator following each trial. In this case, the JH was calculated from the flight time (*t*_*air*_) measured from the vertical ground reaction force:


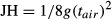


Where *g* = 9.81 m sec^−2^.

All drop jump training was performed with hands held akimbo, and the subjects were instructed to jump to as high as possible with as low contact time as possible.

### Drop height

To standardize the training, the drop height was adjusted for each athlete to achieve a drop velocity of 2.5 m/sec at touchdown. The drop height necessary to attain 2.5 m/sec velocity was found using an infrared grid placed 10 cm above the force platform. The time from passing through the grid to touchdown was used to calculate the velocity at touchdown. The drop height found was subsequently used for both training and tests. The actual drop height varied between subjects from 30 to 36.5 cm, with an average of 33.5 cm. This variation was due to individual differences in takeoff techniques from the starting plateau, which is a well-known phenomenon in studies of drop jumping (Baca 1999). The choice of a drop height of ∼30 cm was based on the experience of Taube et al. (2011), who reported this drop height to be superior to higher heights in a 4-week training regime.

### Pre- and posttraining measurements

During the pre- and posttesting procedures the following parameters were assessed: Jumping performance, maximal isokinetic and isometric muscle strength, kinematics and kinetics of the drop jump movements using a two-dimensional inverse dynamics approach, electromyography (EMG) of muscles in the lower extremities, neural excitability at the spinal level expressed by the Hoffmann (H-) reflex in m. soleus (SOL) and motor output of the soleus motor neurons, the so-called V-wave (Aagaard et al. 2002a).

### Strength measurements

Strength measurements were performed using a Kin-Com dynamometer (Kinetic Communicator, Chattecx Corp., Chattanooga, TN) to obtain measurements of isokinetic and isometric strength of plantar flexors, knee extensors, and knee flexors of the right leg. Isokinetic measurements were performed at an angular velocity of 240°/sec for both concentric and eccentric contractions. For the plantar flexors a range of motion (ROM) of 40° was used, starting at 5° of dorsi flexion. For the knee flexors and extensors a ROM of 10° to 90° of knee flexion was used. On-line visual feedback of the exerted force was provided to each subject on a PC-screen and successive trials were performed in each contraction mode until the subjects were unable to increase peak moment any further. Typically 6–8 trials were performed to fulfill this criterion.

Maximal isometric strength of the right leg was measured at 5° of dorsiflexion for the plantar flexors and 70° of knee flexion for the knee flexors/extensors. The subject was given three trials on verbal countdown to exert as much force, as fast as possible, which was held for 3 sec.

From the latter recordings, rate of force development (RFD) was calculated with respect to the peak force as well as for four time intervals (0–30, 0–50, 0–100, and 0–200 msec) relative to the onset of contraction (Aagaard et al. 2002b). Onset of contraction was defined as the time point at which the force curve exceeded baseline force by 2.5% of peak force.

### Video analysis

The drop jump movement pattern pre- and posttraining was evaluated by assessments of the biomechanics of the right leg. On the pre- and posttraining test days the subjects performed seven drop jumps, which were filmed from the subject's right side using a video camera (JVC GR-DVL9800, Victor Company of Japan, Yokohama, Japan) operating at 120 frames per second. Reflective skin markers were placed on the right side of the body on the fifth metatarsal joint, the lateral malleolus, the lateral femoral epicondyle, the greater trochanter, the anterior superior iliac spine, and on the neck at C5. The drop jumps were performed on two legs (similarly to training) but with the right foot landing on the force platform (AMTI OR6-5-1, Advanced Mechanical Technology, Inc, Watertown, MA) and the left foot on the floor to avoid misleading calculations of center of pressure (COP). Synchronization between video and analog data was obtained by litting a light-emitting diode (LED) in the camera field of view just before the subject left the plateau to jump down on the force platform. At the same time a trigger signal initiated sampling of input from the force platform at 1000 Hz. The whole body center of mass (CM) was calculated from the position data in combination with segmental parameters as described by Winter (1990). The vertical velocity of the CM (*V*_cm_) was calculated by differentiation of the CM position data (*y*-coordinate). *V*_cm_ at the time of takeoff (determined from the vertical ground reaction force signal) was used to calculate the JH:


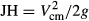


Where *g* = 9.81 m sec^−2^. It is well known that estimation of jumping height during drop jumping is difficult as the energy of the subject at the time of ground contact is unknown (Baca 1999). We considered the *V*_cm_ estimated from the video recordings as the best method for calculation of JH. This is a time consuming method requiring motion capture and therefore not applicable during the training sessions where the flight time was used for calculations of JH as described above.

The six best trials, based on PI, were used for further analysis. The marker position data were low-pass filtered, using a fourth order Butterworth filter with a cutoff frequency of 6 Hz.

Body segment parameters were estimated from marker positions and relation to body mass (Dempster 1955; Winter 1990). Net joint moments about the ankle, knee, and hip were calculated using a 2D inverse dynamics approach (Winter 1990). Power exerted at each joint was calculated as the dot product of joint moment and joint angular velocity.

### Electromyography

EMG was recorded pre- and posttraining (trials separated from the video recordings) from 10 maximal drop jumps from the SOL. The skin was shaved and cleaned with alcohol before mounting the bipolar surface electrodes (Multi Bio Sensors, TX), which were connected to custom build preamplifiers and lead through shielded cables to custom built amplifiers. A common reference electrode was placed on the tibial bone. The electrodes were positioned over the muscle according to Perotto (2005). The signals were sampled at 1000 Hz and stored on a PC for further analysis. EMG recordings of 10 drop jumps were high-pass filtered at 20 Hz, rectified, low-pass filtered at 50 Hz to form linear envelopes (Butterworth fourth-order zero-lag digital filter) and then averaged for each subject.

The preactivity of the muscles was defined as the peak EMG amplitude measured in the time period from 200 msec before touchdown. Furthermore, the peak EMG activity level was measured for the contact phase. The SOL activity was normalized to the maximal M-wave (% M_max_).

### H-reflexes and V-waves of SOL

The site of stimulation of the tibial nerve was carefully located in the popliteal fossa using a probe electrode. Criteria for the optimum site were that (1) the peroneal muscles were not activated by the stimulation and (2) it should preferably be possible to evoke the H-reflex without a detectable M-wave and this was controlled visually before mounting the permanent stimulus electrode. After location of the optimum site, a permanent stimulus electrode (Q-10-A, Medicotest, Ølstykke, Denmark) was placed over the tibial nerve. A 40-mm diameter anode was placed over the patella. The stimulus was a 1 msec square wave pulse delivered by a constant current stimulator (custom built).

**Figure 1:**
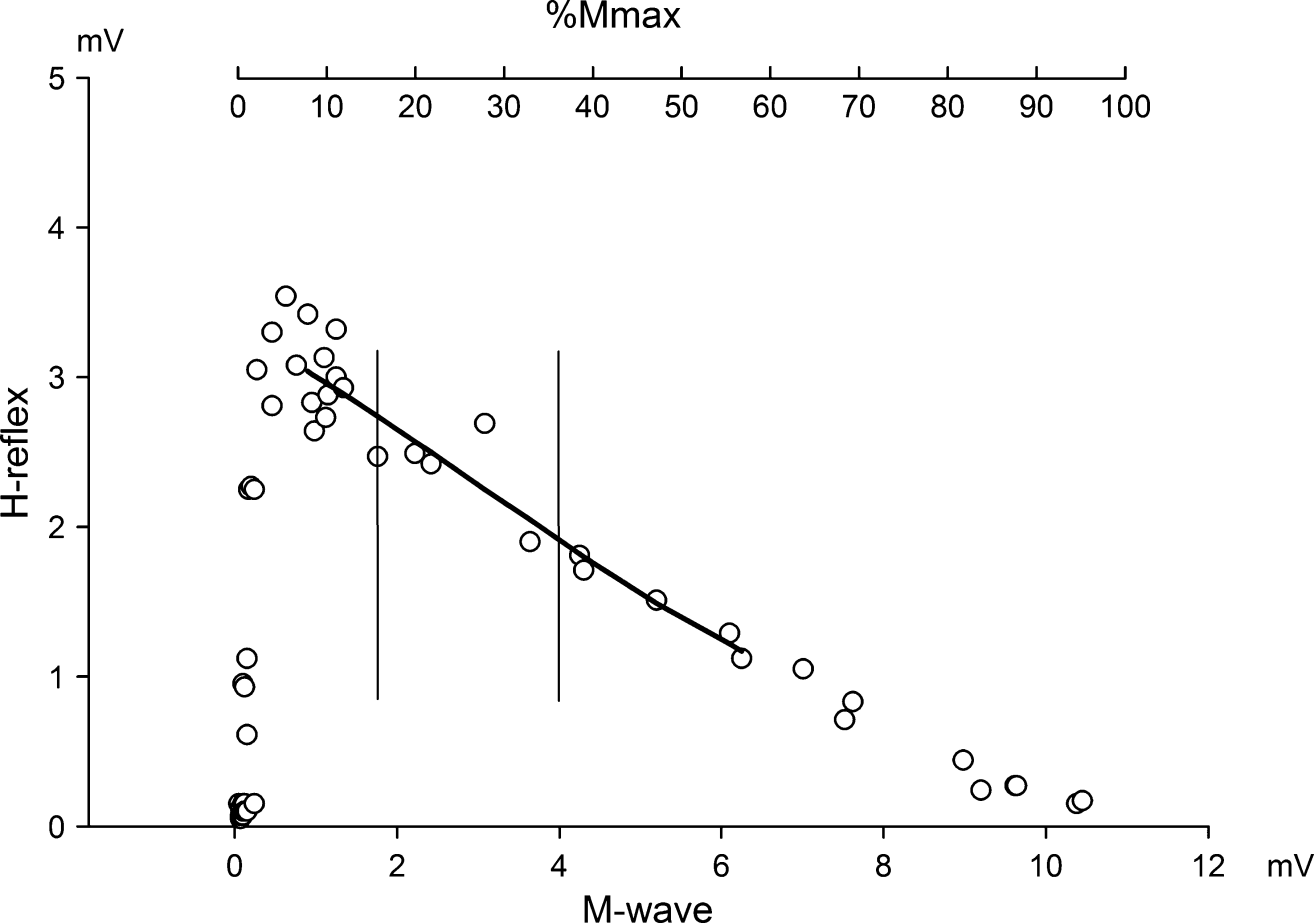
Reference excitability curve of one subject recorded in standing position. A third order polynomium (black curve) was fitted to the descending part of the relation between the M-wave and the H-reflex in the soleus muscle. The stimulus intensity during jumping ranged between 15% and 35% of M_max_ which is indicated by the two vertical lines on the figure. By using a given M-wave amplitude as input an expected H-reflex amplitude (%H_ref_) could be calculated by the polynomial function.

A reference excitability curve of the soleus H-reflex (relation between M-wave and H-reflex) was recorded with the subjects in standing position (Dyhre-Poulsen et al. 1991). A third order polynomial function was fitted to the descending and almost linear part of this relation and later used to normalize the H-reflexes recorded during jumping to the “expected” level during standing (Fig. 1) (Crenna and Frigo 1987; Dyhre-Poulsen et al. 1991). It would be preferable to use the ascending part of the recruitment curve where the H-reflex is more susceptible to changes in spinal excitability. This is, however, not possible during movement as the M-wave has to be used as a measure of the effective stimulus strength. The maximal H-reflex was measured in standing position and expressed relative to the maximal M-wave (H_max_/M_max_).

The stimulator was controlled by a program written in Matlab, which was set to stimulate the tibial nerve at touchdown (i.e., 0 msec), 45 and 90 msec after touchdown, but only one stimulus was applied during each jump. The peak-to-peak amplitude of the M-wave was used as a measure of the effective stimulus strength and only M-waves 15–35% (Fig. 1) of the maximal M-wave (M_max_) were accepted. The H-reflex during jumping was expressed relative to the expected H-reflex (% H_ref_) measured during standing, as defined by the H-M recruitment curve (Fig. 1).

M_max_ was measured in standing position by increasing the stimulus strength until M_max_ did not increase any further. Then during jumping this stimulus strength was doubled to elicit M_max_ for measuring V-waves. The V-wave was expressed relative to M_max_ elicited during jumping.

**Figure 2:**
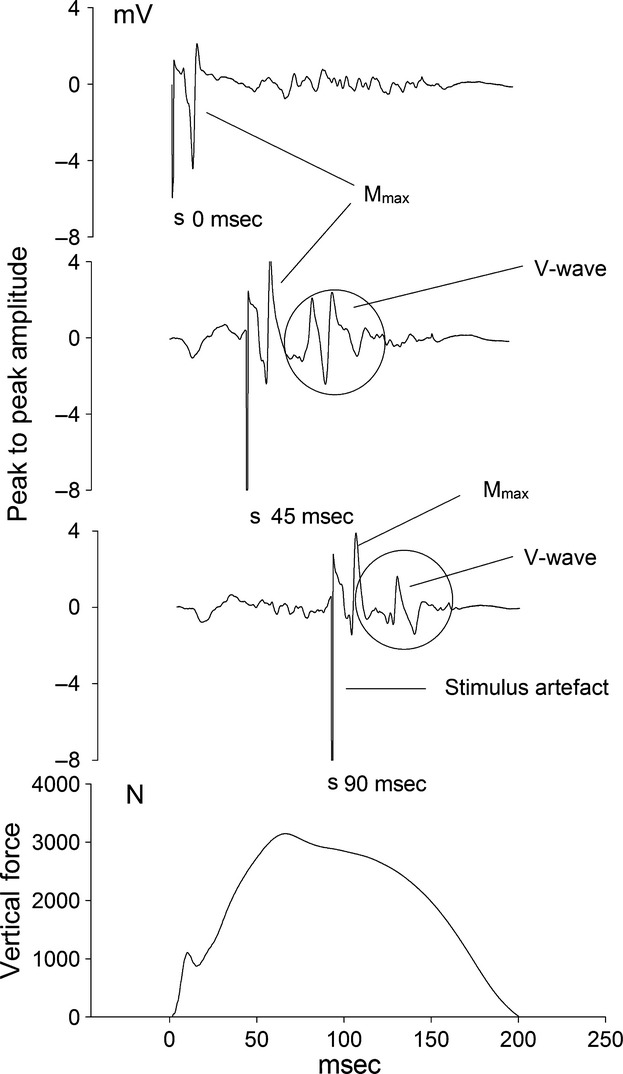
Example of V-waves elicited by a supramaximal stimulus in m. soleus during drop jumps. A large stimulus artifact is visible about 10 msec prior to the maximal M-wave. From top to bottom: (1) the stimulus elicited at touchdown (0 msec) resulting in a very small V-wave, if any, (2) the stimulus elicited 45 msec after touchdown with a clear V-wave, (3) the stimulus elicited after 90 msec also with a clear V-wave, (4) the vertical ground reaction force. All curves are averaged from five trials of a representative subject.

The V-wave can only be elicited during voluntary activity of the muscle in question, in this case the soleus muscle. It is elicited by a supra maximal stimulus, which produces a maximal M-wave, which should normally cause the H-reflex to be extinguished by collision of antidromic and orthodromic action potentials in the efferent axons of the tibial nerve a few centimeters outside the spinal cord (Fig. 1). However, extensive voluntary activity conveyed to the motor neurons of the soleus muscle through corticospinal pathways is likely to collide with the antidromic potentials from the stimulus and thereby clear the way for a variation of the H-reflex, in this case called the V-wave (Fig. 2) (Sale et al. 1983; Aagaard et al. 2002a). An increased V-wave after a training period may be due to either increased excitability of the motor neurons, which can be measured by the H-reflex and/or increased input to the motor neurons from, for example, descending pathways.

In one subject the H-reflex could not be measured reliably. The H-reflex and V-wave results are therefore based on eight subjects.

### Statistical analyses

Data were expressed as group mean values ± SE. Differences in jumping performance, muscle strength, muscle activity, biomechanical, and reflex response parameters between pre- and posttraining were tested by a paired Students *t*-test (two tailed). The within-subject variability in jumping performance was assessed by the coefficient of variation (CV) calculated by dividing the SD of the JH by the mean JH observed in each training session for each subject. A one way analysis of variance test (ANOVA) for repeated measures was used to evaluate the jumping performance over the 4 weeks of training. Data from eight subjects obtained during the training intervention was reduced to five parameters defined as means of drop jumps performed at the first training session (1. train.), means of drop jumps performed during training sessions 2–3 (week 1), 4–6 (week 2), 7–9 (week 3), and 10–12 (week 4). In case of significant main effects, post hoc tests were performed as the Duncan's method. The level of significance was set at 5%.

## Results

### Jumping performance before and after the training period

The jumping performance was significantly improved after 4 weeks of drop jump training (Table 1). The drop jumping height was significantly increased by 11.9% (*P* = 0.024). The PI increased significantly by 16.2% (*P* = 0.009) (Table 1). However, the contact time remained unchanged after training indicating that the improvement of the PI was primarily explained by an increased jumping height (Table 1).

**Table 1:** Summary of jump performance

	Jump height (cm)	Contact time (msec)	PI (m/sec)
Pre	29.21 ± 1.23	181.2 ± 10.58	1.66 ± 0.11
Post	32.69 ± 1.39	172.5 ± 5.93	1.92 ± 0.11
*P*-value	0.024	0.109	0.009

Values are mean ± SE.

### Jumping performance during the training period

**Figure 3:**
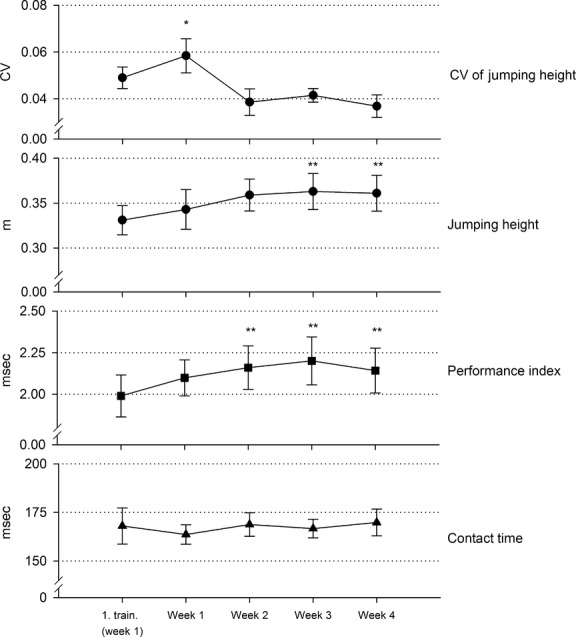
Progression of jumping performance during training (*n* = 8). From top: Coefficient of variation (CV) of the jumping height, jumping height (m), performance index (m/sec), contact time (msec). Values are means ± SE. Jumping performance over the 4 weeks are means of drop jumps performed at the first training session (1. train.), training sessions 2–3 (week 1), 4–6 (week 2), 7–9 (week 3), and 10–12 (week 4). Results in this figure are based on 1447 drop jumps recorded during the training intervention in eight out of nine subjects (see text for further information). One way ANOVA for repeated measures showed a statistical significant difference for CV of the jumping height (*P* = 0.019), jumping height (*P* = 0.026), and performance index (*P* = 0.009). Asterisks indicate statistical significant differences located by the post hoc pairwise comparisons: *significantly different from week 2, 3, and 4 (*P* < 0.01), **significantly different from 1. train. and week 1 (*P* < 0.05).

In general, the jumping performance assessed by CV of jumping height, JH, PI, and contact time reflected a learning effect over the 4 weeks of training (Fig. 3). The JH and PI changed significantly during the training period, while no significant changes were observed in the contact time (Fig. 3). From week 2 a plateau effect in the JH, PI, and CV of JH was observed, although JH and PI were still significantly higher at the end of training compared to the prevalues (Fig. 3). In addition, the variation in JH decreased significantly during the last part of the training period (week 2–4) where the CV of the JH on average was reduced by 33.3%, when compared to week 1 (*P* < 0.010) (Fig. 3).

### Biomechanical parameters

**Figure 4:**
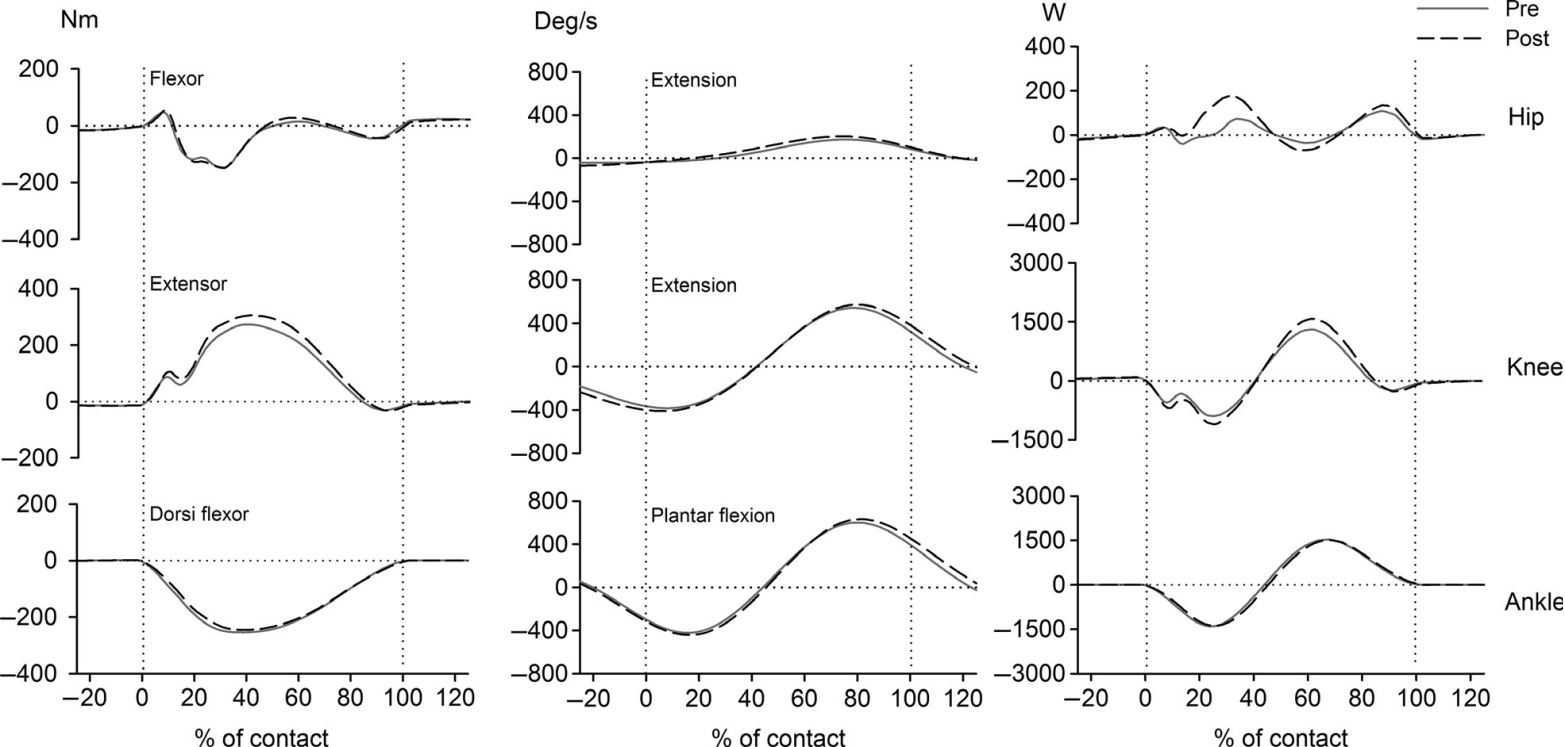
Average drop jump biomechanics of all subjects (*n* = 9) pretraining (solid gray) and posttraining (dashed black). Left from top to bottom: Net joint moments about the hip, knee, and ankle joint (Nm). The middle panel shows angular velocity (deg/sec) about the same joints while the right panel shows muscle power (W) about the hip, knee, and ankle joint. Positive power indicates concentric muscle work and negative power eccentric muscle work about the joint. On the *x*-axis, 0–100% represents the contact phase.

In general, the drop jump movement time course pattern did not change significantly after training (Fig. 4). Following training, there was a clear tendency toward an increase in the peak knee extensor moment and peak concentric knee joint power. These differences were, however, insignificant (Fig. 4, Table 2). No changes in the biomechanics for the hip and ankle joints were observed (Table 2). However, the sum of the ankle and knee peak concentric power increased significantly after training by 7.1% (Table 2). After training seven subjects showed increased peak values of the knee extensor moment and power, while the two remaining subjects showed increased peak values regarding biomechanics of the ankle plantar flexors. Clearly, the ankle plantar flexors and knee joint extensors dominated the drop jump takeoff phase as concentric power generators, while the role of the hip joint extensors appeared to be modest (Table 2, Fig. 4).

**Table 2:** Summary of biomechanical parameters pre and post 4 weeks of drop jump training

	Peak joint moments (Nm)			Angular velocity (deg/sec) – concentric phase^1^			Joint power (W) – concentric phase^1^		
			
	Pre	Post	*P*-value	Pre	Post	*P*-value	Pre	Post	*P*-value
Ankle	−261.5 ± 22.5	−253.1 ± 19.9	0.624	610.9 ± 14.3	637.2 ± 18.5	0.199	1580.8 ± 131.4	1542.71 ± 89.8	0.759
Knee	284.6 ± 21.4	330.2 ± 23.2	0.076	551.4 ± 25.9	576.6 ± 15.0	0.270	1375.0 ± 117.0	1621.5 ± 102.5	0.059
Hip	−61.4 ± 26.8	−59.3 ± 39.5^2^	0.940	177.4 ± 25.1	208.3 ± 17.1	0.090	−27.4 ± 42.2	−46.1 ± 46.8^2^	0.503
Ankle + Knee^3^	–	–	–	–	–	–	2955.8 ± 170.6	3164.2 ± 144.3	0.047

Values are mean ± SE.

1 Peak angular velocity and joint power values were measured in the concentric phase corresponding to second half of the contact phase (see Fig. 4).

2 The hip joint moment and power values were measured at the same time where the knee joint moment and concentric knee joint power peaked, respectively.

3 The sum of the peak knee and ankle joint power.

### Muscle activities and reflex responses

**Figure 5:**
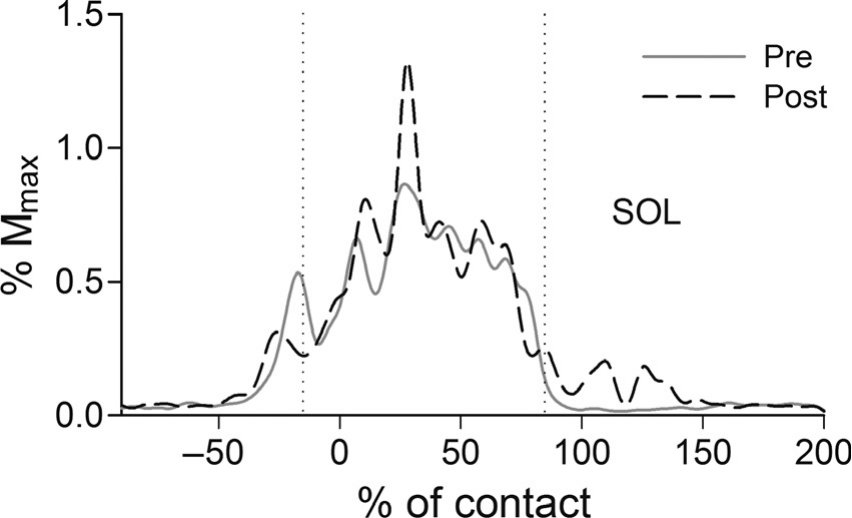
Average linear envelopes of m. soleus (SOL) based on 10 maximal drop jumps pretraining (solid gray) and posttraining (dashed black) from one representative subject. On the *x*-axis, 0–100% represents the contact phase.

**Figure 6:**
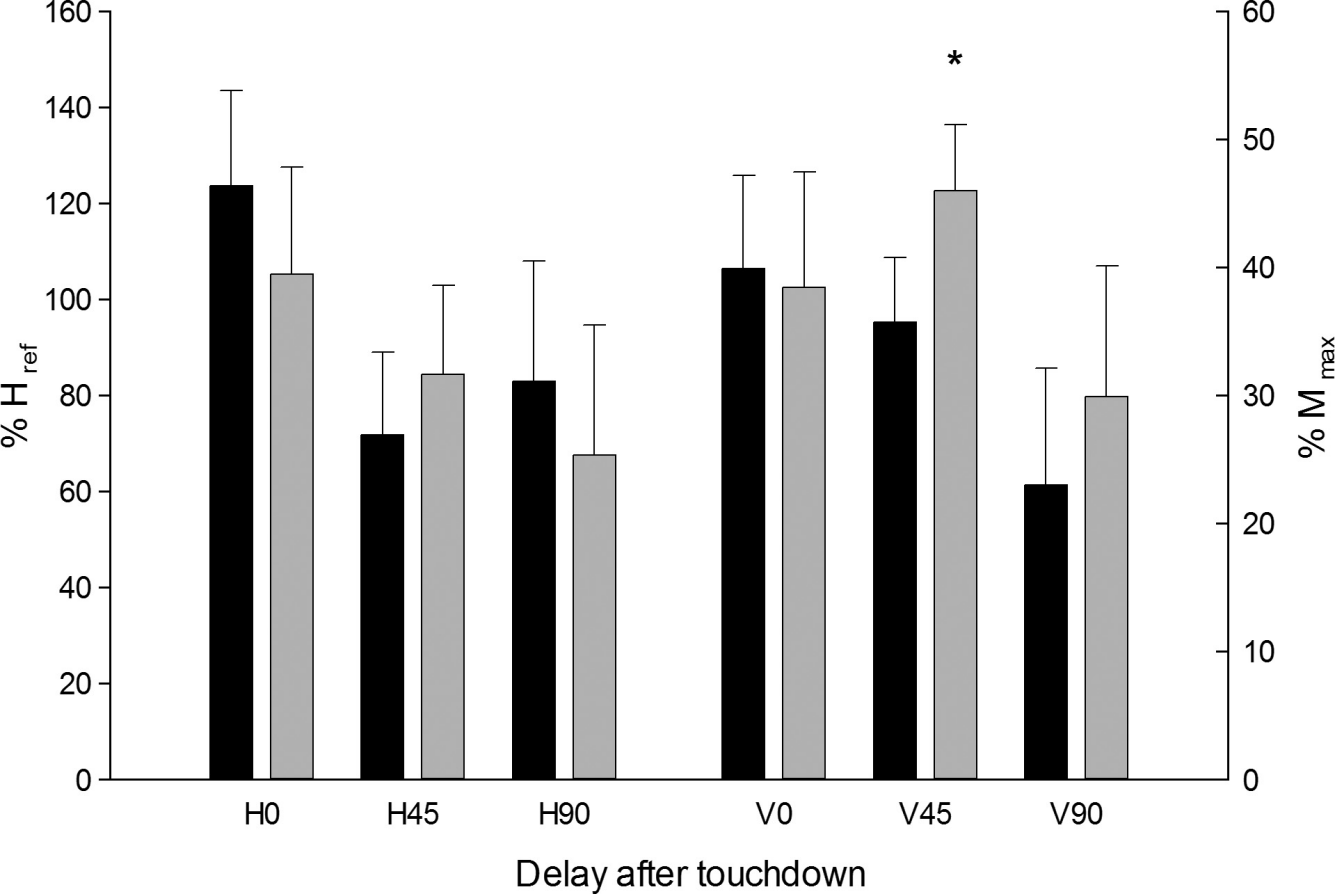
H-reflex and V-wave amplitudes in m. soleus before (black bars) and after (gray bars) training. The stimuli were elicited at touchdown (H0, V0), 45 msec after touchdown (H45, V45), and 90 msec after touchdown (H90, V90). The H-reflex amplitude was expressed relative to the expected H-reflex (%H_ref_) measured in standing position (see Fig. 1). The V-wave was expressed relative to the maximal M-wave measured during jumping. Bars are averaged from eight subjects. Error bars are SE. The V-wave elicited 45 msec after touchdown was significantly higher after the training period, which is indicated by an asterisk.

The peak preactivity in the SOL muscle prior to touchdown decreased 16% after training (*P* = 0.015) (Fig. 5). No other differences in the EMG results were observed after training. The H_max_/M_max_ ratio measured in standing position was unchanged after the training period. A typical reference excitability curve is seen in Figure 1. In general, a rather high H-reflex excitability of ∼120% H_ref_ measured in standing position was observed at touchdown, while the amplitudes measured after 45 and 90 msec were somewhat lower (Fig. 6). However, no changes were observed in the H-reflex after the training period (Fig. 6).

A typical series of sweeps containing the maximal M- and V-waves are illustrated in Figure 2. The V-wave was often higher than the H-reflex (Fig. 6). After 4 weeks of training a significant increase of 28.8% was observed in the V-wave elicited 45 msec after touchdown (*P* = 0.044) (Fig. 6). No changes were observed at touchdown or after 90 msec.

### Muscle strength

Maximal isometric and isokinetic muscle strength as well as RFD of the ankle plantar flexors and knee flexors and extensors were unchanged posttraining.

## Discussion

The key findings of this study were that the jumping performance improved significantly during and following 4 weeks of intensive drop jump training in well-trained athletes with a high level of jumping experience. With no improvements in basic strength measurement, the results suggest that the improved jumping performance is explained by an optimization of the neuromuscular activation pattern controlling the drop jump movement. In the present study, a wide number of biomechanical and neurophysiological measures were obtained before and after the training intervention. We believe that these results have provided new insight in the mechanisms responsible for the improved drop jump performance. Furthermore, as the training was closely monitored in eight out of nine subjects, it was possible to determine effects of motor learning. Although the participants in the present study were skilled jumpers a learning effect was observed for the jumping performance during the course of training. A plateau effect in performance occurred during the second week of training (Fig. 3). In addition, the jumping performance became more consistent during the last part of the training period (Fig. 3) where the variation in jumping height was significantly reduced. This decreased variation together with the improved performance reflects a motor learning effect (Cohen and Sternad 2009). Given that we observed no changes in muscle strength it is highly likely that the decreased variation and improved performance are explained by an improvement of muscle activation patterns (i.e., coordination) following training. Finally, it is possible that the results reflect consolidation effects and that the improved motor performance may be retained in the future (Robertson et al. 2004). Following the training period, the JH and PI improved by 11.9% and 16.2%, while the contact time remained unchanged (Table 1, Fig. 3). Thus, the increased PI was primarily explained by the increased JH. It was imperative that the drop jumps were performed with a very short contact time. It is possible that the combination of skilled jumpers and drop jumps performed with very short contact explains why the contact time was unchanged posttraining. No control group was included in the present study which prevents us from quantifying the true effect of training and the results presented in Figure 3 shows the combined effect of testing and training. However, it was impossible to recruit control subjects who matched the level of performance and jumping experience as characterized by the study group.

The vertical jumping height depends both on the capacity of the leg muscles to generate energy and on the coordination of the muscle activations, that is, ability of the sensorimotor system to ensure that the maximal amount of the released leg muscle energy is used to accelerate the body CM vertically (Bobbert and van Ingen Schenau 1988; Bobbert 1990). The present results indicate that the subjects improved the drop jump performance by a combined increase of the peak concentric knee and ankle joint power output. In seven out of nine subjects the knee joint power was increased after training, while in two subjects increased ankle joint power was observed. Thus, adaptations to 4 weeks of drop jump training seem to be differentiated between individuals, and individual jumping strategies may exist among these skilled jumpers.

The biomechanical results clearly showed that the knee extensors and ankle plantar flexors were the dominating muscles during the drop jump takeoff phase, while the hip joint played a minor role here. The hip joint power was generally low, about 1/10 compared to the knee and ankle joint. It is possible that the hip joint muscles primarily are used to maintain balance of especially the upper body rather than contributing to jumping height. During the second half of the contact phase a negative peak in the hip joint power was observed (Fig. 4, Table 2), which obviously appears to work against the knee and the ankle joint during push off. It is unlikely that a large hip extensor moment producing positive power in this phase would enhance vertical drop jump performance when the contact time is very short but rather lead to a backwards somersault instead of a vertical jump with takeoff and touchdown in the same place.

It has been reported that preactivity is important to improve drop jump performance (McBride et al. 2008), but in the present study preactivity in the soleus muscle decreased significantly after training. It is likely that athletes at this performance level are able to fine-tune both preactivity and the level of cocontraction between the ankle plantar and dorsiflexors to an optimum.

It is often said that the H-reflex is a measure of motor neuron excitability, but to be more precise the H-reflex is a measure of the transmission from Ia afferents to the α-motor neurons (Schieppati 1987), that is, transmission in the central part of the monosynaptic stretch reflex circuitry. This transmission is dependent on presynaptic inhibition as well as postsynaptic factors, that is, motoneuron properties, inhibition, and facilitatory effects. The soleus H-reflex has been shown to increase after a period of jumping training (skipping) (Voigt et al. 1998). However, in the present study, the amplitude of the soleus H-reflex was unchanged after training both during standing and during jumping. This may imply that the drop jump training period was not accompanied by significant changes in the transmission in the monosynaptic stretch reflex circuitry, that is, in presynaptic inhibition or changes in spinal motor neuron properties. Furthermore, Voigt et al. (1998) included healthy subjects and not well-trained and skilled jumpers as in the present study, which also may explain the different observations.

The V-wave 45 msec after touchdown increased significantly after training. This indicates an increased net excitation of the motor neuron pool which could originate from descending pathways, that is, the corticospinal tract or from interneurons in the spinal cord. An increased excitation may have caused an increased firing frequency of the motor neurons to the soleus muscle. However, this is not likely to have had an effect on the motor units of the soleus muscle as these are generally small and contain type I slow twitch muscle fibers. These fibers are already tetanized at 8–10 Hz (Bigland and Lippold 1954; Duchateau and Enoka 2011) so it will not help to innervate them at higher frequencies. However, the quadriceps muscle contains many large motor units consisting of fast twitch fibers, which may benefit from firing rates up to about 30–40 Hz to produce more force (Bigland and Lippold 1954; Duchateau and Enoka 2011). The gastrocnemius muscles contain also large motor units, but the two gastrocnemii together are only about 1/5 in physiological cross-sectional area compared to the soleus (Wickiewicz et al. 1983). As the soleus and the quadriceps are so-called homonymous muscles whose Ia afferents project onto both muscle's motor neuron pools, it could be speculated that the firing frequency may also have increased in the quadriceps, which would contribute to improved function of the knee joint extensors posttraining. In support of this, the knee extensor moment and power output was improved in seven out of nine subjects after training.

In conclusion, 4 weeks of intensive supervised drop jump training from moderate drop height was capable of inducing a significant increase in maximal jumping height in trained athletes without any measurable increases in isometric or isokinetic muscle strength or RFD, which confirms our initial hypothesis that improved jumping performance would be due to neural and not morphological factors. We suggest that the improved concentric power output generated by the ankle and knee joint extensor muscles observed after training was due to neural factors. The increased V-wave observed 45 msec after touchdown supports that the improved jumping performance was caused by changes in neural control of the movement. We suggest that the improved jumping performance was due to an optimization of the coordination and activation pattern controlling the drop jump movement enabling improved jumping performance and a smaller variability, that is, enhanced consistency of the motor output.
